# Impact of residual covariance structures on genomic prediction ability in multi-environment trials

**DOI:** 10.1371/journal.pone.0201181

**Published:** 2018-07-20

**Authors:** Boby Mathew, Jens Léon, Mikko J. Sillanpää

**Affiliations:** 1 Institute of Crop Science and Resource Conservation, University of Bonn, 53115 Bonn, Germany; 2 Department of Mathematical Sciences and Biocenter Oulu, FIN-90014 Oulu, Finland; Institute for Resistance Research and Stress Tolerance, GERMANY

## Abstract

In plant breeding, one of the main purpose of multi-environment trial (MET) is to assess the intensity of genotype-by-environment (G×E) interactions in order to select high-performing lines of each environment. Most models to analyze such MET data consider only the additive genetic effects and the part of the non-additive genetic effects are confounded with the residual terms and this may lead to the non-negligible residual covariances between the same trait measured at multiple environments. In breeding programs it is also common to have the phenotype information from some environments available and values are missing in some other environments. In this study we focused on two problems: (1) to study the impact of different residual covariance structures on genomic prediction ability using different models to analyze MET data; (2) to compare the ability of different MET analysis models to predict the missing values in a single environment. Our results suggests that, it is important to consider the heterogeneous residual covariance structure for the MET analysis and multivariate mixed model seems to be especially suitable to predict the missing values in a single environment. We also present the prediction abilities based on Bayesian and frequentist approaches with different models using field data sets (maize and rice) having different levels of G×E interactions.

## Introduction

Genomic selection (GS) [1] has revolutionized both animal and plant breeding programs with the advances in new genotyping technologies. In GS, the selection decisions are based on the genomic estimated breeding values (GEBVs) which are calculated based on genome-wide dense set of markers. In both animal and plant breeding, GS will accelerate genetic gain for various complex traits. Increased genetic gains using GS have been already reported by many studies in crops [2] as well in livestock [3] breeding programs.

In plant breeding, multi-environment trials (MET) are mainly used for two breeding purposes: 1) to find the stable high-performing lines—main GEBVs—across environments, 2) to find the most adapted superior genotype—specific GEBV—for a specific region. If genomic prediction is performed in the first scenario, different environments can be treated as a sample from a Target Population of Environments (TEP) [4] and GEBVs can be estimated across environments by considering the main effects across environments (for exception see [5]). However, in the second scenario, the aim is to find the locally adapted genotype and the prediction models which consider only main effect may limit their predictive power/accuracy by ignoring G×E interaction term in the model. Thus, if the breeding target of MET is to find the most adapted genotypes of each environment, it is essential to consider G×E interactions while estimating GEBVs. Additionally, the presence of the G×E interactions are expected to have negative impact on the accuracy of GP when the environments are significantly different for the training and validation genotypes [6, 7].

When the aim of MET analysis is to find the best performing lines for a specific environment, different approaches have been proposed to tackle the G×E interactions. A common approach is to consider a single phenotype measured at multiple environments as different correlated traits and analyze them using a multivariate modeling framework. Genotype-by-environment interactions can also be tackled with a two-way mixed model fitted with an own random effect and covariance matrix for G×E effects. The Factor-analytic (FA) model is also commonly used to analyze MET data and have been extensively studied (*e.g.*, [8–11]).

Most of the current models for genomic prediction of MET data are based on frequentist inference (*e.g*, [5, 7, 12–15]). Recently some studies applied Bayesian variable selection models [16–18] and Bayesian Gaussian kernel model [19] for the genomic prediction in MET data. However, the studies using Bayesian GBLUP for genomic prediction in MET data are limited.

Most of the univariate and multivariate genomic prediction models consider only the additive genetic effects and are thus based on the additive genomic relationship matrix (note that there has been studies [19, 20] also to model non-additive genetic effects in genomic prediction with MET data). In such models part of the non-additive genetic effects are confounded with the residual terms and this may lead to the non-negligible residual covariances between the same trait measured at multiple environments [21, 22]. Thus it is important to take into account such residual covariances in the prediction models for MET data as emphasized in [13]. The multivariate model allows more flexible handling of covariance structures for the residuals. One of important characteristics of MET is that often the trait measurements from a single environment may be missing.

Motivated by this, we want to study the impact of different residual covariance structures as well as missing data patterns on genomic prediction ability using a multivariate mixed model especially in Bayesian GBLUP framework. For that we use two real MET data sets of rice and maize having the phenotypic observations collected from three different environments and showing different levels of G×E interactions. The main reason to select these datasets were that the rice dataset showed strong genomic correlation between environments (less G×E interactions), whereas the maize showed strong G×E interactions. Thus, we could also study the impact of varying levels of G×E interactions on genomic prediction abilities with different models to analyze MET data, to find the most adapted lines of each environment. We also report the prediction abilities based on frequentist estimation methods along with the Bayesian approach. Additionally as for comparison purposes we also present the results from the univariate mixed model and G×E interaction mixed model.

## Materials and methods

In order to study the impact of residual covariance structures on genomic prediction ability, we considered three different models in mixed model framework. In the first model we assumed the trait measured in different environments are correlated and analyzed them using a multivariate mixed model. In the second model we considered genotype by environment interaction as a second random term in the model along with the additive genetic effect. This model provide estimates of genetic effects along with the G×E interaction effects. In the third model we considered the phenotypic information from each environment separately (no location effect assumed in the model). The three models are explained in the following section.

### Model 1: Multivariate mixed model

Let us consider a single trait measured from the same individual at multiple environments as separate traits. We want to analyze such multi-environmental trial of a single trait using the multivariate mixed model. Let us consider **l** different environments/locations so that the vector **y**_1_ contain **n** observations from the first environment, **y**_2_ that of the second environment, and **y**_**l**_ the observations from the **l**^**th**^ environment. Then the multi-environmental mixed linear model for **l** locations can be written as:

$$\begin{array}{c} y_{i}=X_{i}\beta_{i}+Z_{i}u_{i}+\epsilon_{i},\;i=1,2..,l \end{array}$$ (1)

Here **β**_**i**_ is a vector of fixed effects associated with environment *i*, **u**_**i**_ is a vector of random additive genetic effects associated with the environment *i* (note that the genotype by environment interaction effects are confounded with the main genetic effect and may differ between locations), **ϵ**_**i**_ is a vector of error terms associated with the environment. Moreover, **X**_**i**_ and **Z**_**i**_ are known incidence matrices for the fixed effects and the random effects for the location *i*, respectively. In our study we considered the phenotypic observations from three locations so *i* = 1, 2, 3. Thus $\beta ={\left[\beta_{1}^{{}^{\prime }},\beta_{2}^{{}^{\prime }},\beta_{3}^{{}^{\prime }}\right]}^{{}^{\prime }}$, **u** = [**u**^**′**^_**1**_, **u**^**′**^_**2**_, **u**^**′**^_**3**_]′, **ϵ** = [**ϵ**^**′**^_**1**_, **ϵ**^**′**^_**2**_, **ϵ**^**′**^_**3**_]′ and **y** contains the phenotypic observation from the locations **y**_1_, **y**_1_, **y**_3_. Then mixed model equation (MME) for the model (1) is:

$$\begin{array}{c} \left[\begin{array}{cc} X^{\prime }R^{-1}X & X^{\prime }R^{-1}Z\\ Z^{\prime }R^{-1}X & Z^{\prime }R^{-1}Z+G^{-1} \end{array}\right]\left[\begin{array}{c} \beta \\ u \end{array}\right]=\left[\begin{array}{c} X^{\prime }R^{-1}y\\ Z^{\prime }R^{-1}y \end{array}\right]. \end{array}$$ (2)

Here, **R** and **G** are covariance matrices associated with the vector **ϵ** of errors and vector **u** of random effects. If **R**_0_ (of order 3 × 3) is the residual covariance for the three locations then **R** can be calculated as **R** = **R**_0_ ⊗ **I** ((here ‘⊗’ is the Kronecker product of two matrices and **I** is the identity matrix). Similarly, the genetic covariance matrix **G** can be calculated as **G** = **G**_0_ ⊗ **K**. Here **K** is the additive genomic relationship matrix which was calculated based on the available marker information following the first approach of VanRaden method [23] and **G**_0_ is a 3 × 3 genomic covariance matrix.

For the Bayesian inference using model (1) one need to specify the conditional distribution for the data (**y**) and prior distribution for the unknown parameters. So the conditional distribution of data **y**, given the parameters assumed to follow a multivariate normal distribution:

$$\begin{array}{c} y|\beta ,u,R_{0}∼N\left(X\beta +Zu,R_{0}⊗I\right). \end{array}$$ (3)

The additive genetic effects (***u***_*i*_’*s*) were assigned multivariate normal distributions with a mean vector of zeros, **0**, as:

$$\begin{array}{c} u|G_{0},K∼N\left(0,G_{0}⊗K\right), \end{array}$$ (4)

and the errors (***ϵ***_*i*_’*s*) were assumed to follow,

$$\begin{array}{c} \epsilon |R_{0}∼N\left(0,R_{0}⊗I\right), \end{array}$$ (5)

where **I** is an identity matrix. In Bayesian analysis fixed effects also have a prior and here **β** was assigned a vague, large-variance Gaussian prior distribution.

In order to study the effect of different homogeneous and heterogeneous residual covariance structures to the GEBV estimation accuracy, we considered following structures for **R**_0_. 1) The first-order ante dependence (ANT1) covariance structure, which allows unequal variances over different locations and unequal correlations and covariances among different locations, 2) The unstructured (US) covariance structure, which allows unequal variances over different locations and unequal covariance between different locations, 3) The diagonal homogeneous covariance (IDV) structure, which allows a constant variance across all locations and 4) The diagonal heterogeneous covariance (IDH) structure, which allows different variances across different locations. The ANT1 structure requires *l* + (*l* − 1) parameters (*l* is the number of locations) to be estimated, whereas the US requires the estimation of *l*(*l* − 1)/2 parameters (see [24] for more details). Additionally, the inverse of the ANT1 covariance structure is tri-diagonal (only three diagonals are non zero) and has less parameters to be estimated than the US covariance structure when *l* is greater than three. However, for the genomic covariance matrix (**G**_0_), we assumed a *priori* the unstructured covariance structure in all the cases.

### Model 2: G×E interaction mixed model

In order to model the genotype by environment interaction we considered the following mixed model:

$$\begin{array}{c} y=X\beta +Zu+Wv+\epsilon \end{array}$$ (6)

**β**_**i**_ is a vector of fixed effects (in this case including grand mean and location effects), **u**_**i**_ is a vector of random additive genetic effects, **v** is the vector of random genotype by environment interaction effects, **ϵ** is a vector of error terms associated with the locations. Moreover, **X** and **Z** are known incidence matrices for the fixed effects and the random additive genetic effects, respectively. Here, the dimension of **X** is (*l* × *n*) × (1 + *l*) (1 for the grand mean, *l* is the number of locations and *n* is the number of lines) and **Z** is (*l* × *n*) × (*n*). Finally, **W** is the incidence matrix which relates the lines to different locations. In our study we considered three locations (1, 2, 3) thus: **ϵ** = [**ϵ**^**′**^_**1**_, **ϵ**^**′**^_**2**_, **ϵ**^**′**^_**3**_]′, which are independently normally distributed with mean zero and variance $\sigma_{e}^{2}$ ($\epsilon_{i}∼N\left(0,\sigma_{e}^{2}I\right)$), and **y** = [**y**^**′**^_**1**_, **y**^**′**^_**2**_, **y**^**′**^_**3**_]′. Moreover $u|K∼N(0,\sigma_{g}^{2}K)$, $v|K∼N(0,\sigma_{v}^{2}I⊗K)$. Here **K** is the additive genomic relationship matrix calculated using the available marker information and **I** is a **3** × **3** identity matrix. With this model the GEBVs were estimated as **u** + **v**_*l*_ (here **v**_*l*_ is location specific interaction effect). Model 2 can be seen as a special case of model 1 with **R**_0_ being IDV structure and **G**_0_ having identical diagonals and identical off-diagonals (thus two parameters to be estimated). This model is commonly known as the compound symmetry model [25]. Additionally, with model 2 it is also possible to model the heterogeneous residual covariance structure (IDH).

### Model 3: Univariate mixed model

We also performed univariate analysis using the phenotypic information from a single location and for that we considered the following model:

$$\begin{array}{c} y=X\beta +Zu+\epsilon \end{array}$$ (7)

with $u|K∼N(0,\sigma_{g}^{2}K)$ and $\epsilon ∼N(0,\sigma_{e}^{2}I)$. Here **y** is the phenotypic information from a single location and **K** is the additive genomic relationship matrix calculated using the available marker information. Moreover, with model 3 the dimension of the incidence matrix **X** is *n* × 1 (1 for the grand mean) and for **Z** is *n* × *n* (*n* is the number of lines).

### Example datasets

In order to compare the different models we used two real dataset having the phenotypic information from three different locations.

**Rice dataset:** This dataset is publicly available at http://www.ricediversity.org/data/ and consists of 413 diverse accessions of O. sativa [26]) collected from 82 different countries. The accessions were genotyped with single nucleotide polymorphism (SNP) markers and 36 901 SNPs were available for the analysis after excluding markers with minor allele frequency (MAF) ≤ 0.05 and missing values ≥ 20%. [26] measured the trait flowering time in three different locations. The first location (ARK) was in Stuttgart, Arkansas, USA, the second one in in Aberdeen (ABR) and the third location was Faridpur (FAD), Bangladesh (see [26] for more details). Out of the 413 lines, phenotypic informations were missing for 42 lines in all three environments and we did not consider those for the final analysis. So we analyzed a subset of 371 lines in this study.

**Maize dataset:** This data set consists a total of 504 double-haploid maize lines and the phenotype as well as the genotype information which are all made publicly available [15]. Three traits, yield (Yield), anthesis-silking interval (ASI) and plant height (PH) were measured in three rain fed environments called E1, E2 and E3 (see [15] for more details about the experimental design). This dataset was genotyped using genotyping-by-sequencing (GBS) method and after filtering for the minor allele frequency, around 158 281 SNPs were available for the analysis. One of the main problem with GBS is the large proportion of missing genotypes and in order to cope with that, while calculating the additive genomic relationship matrix, Crossa et al [15] modified the method of VanRaden [23] to account for the missing genotypes (see [15] for more details). In this study we analyzed the trait Yield (which was already standardized to unit variance). This dataset consist of markers whose MAF was ≥ 0.05 and the markers that had maximum of ≤ 20% missing values. For both data sets (Rice and Maize) pedigree information was not available.

### Cross validation (CV)

We applied five fold cross validation [27] in order to estimate the prediction abilities of different mixed models using the real datasets. Prediction abilities were calculated as the Pearson correlations between the observed and predicted phenotypes (GEBV). We repeated the five fold CV procedure 10 times and the prediction ability estimates were averaged over to produce a single estimate. In five fold CV, we used 80% of the data as the training set and the remaining 20% as the validation set (due to computational challenges we only considered five fold cross validation (80/20), however it might be interesting to estimate the prediction abilities using other combination like 70/30, 60/40, and 50/50 by reducing the training population size). We used the same training and validation sets in each analysis. For the cross validation using models to tackle G×E effects (model 1 & 2), following [13] we used two different approaches, with the first approach, in the validation set we included the lines from all three environments and obtained genomic prediction abilities. In the second approach we selected the lines from only a single environment into the validation set (note that the phenotype information from the other two environments were included in the training set) and calculated the prediction ability for that single environment. The second approach was intended to mimic the situation where the breeder has the phenotype from two environments available and a value missing in the third environment. Hereafter we refer the first approach as multiple environment cross validation (M_CV) procedure and the latter one as single environment cross validation (S_CV) procedure. Finally, we also applied the CV to estimated the prediction abilities using the univariate model (model 3) assuming normal distributed random residuals and refer as RND.

### Estimation

In order to compare the prediction abilities we estimated the Bayesian and traditional GBLUP using all three models. For the Bayesian analysis we used the R package *’MCMCglmm’* [28], which is based on Markov Chain Monte Carlo (MCMC) sampling methods. We considered a total MCMC chain of length 10 000 iterations with a burning period of 3 000 iterations for the Bayesian inference with the multivariate as well as the univariate model and calculated the posterior mode of the distribution. In multivariate GBLUP estimation using *MCMCglmm* package we assigned inverse-Wishart with a diagonal scaling matrix (the diagonal elements were the univariate variance components estimate corresponding to each location) as the prior distribution for the random genetic (**G**_0_) and residual (**R**_0_) covariance matrices between the three locations. The traditional multivariate GBLUP estimation was performed using the recently published R package *’sommer’* [29]. Using *MCMCglmm* we were able to estimate parameters from model 1 using all the four residual covariance structures, but with *sommer* we were able to consider only the IDH and US covariance structures.

## Results

### Rice data

Our univariate analysis showed strong genomic correlation between the environments and the genomic correlation based on univariate and multivariate analysis are shown in Table 1 along with the SNP heritability estimates. Here the narrow-sense SNP-heritabilities (*h*^2^) were estimated as: *h*^2^ = $\sigma_{g}^{2}/(\sigma_{g}^{2}+\sigma_{e}^{2}$), here $\sigma_{g}^{2}$ and $\sigma_{e}^{2}$ are the genomic and residual variances, respectively. It is not a common practice to estimate the genomic correlation between locations based on univariate model by considering a single environment, but for the comparison point we present the results here. The genomic correlation between the environments were higher for the multivariate approach compared to the univariate model. Due to the strong genomic correlation between the environments. For both M_CV and S_CV cross validation procedures using model 1, the US, IDH and ANT1 residual covariance structures showed similar prediction abilities, whereas the prediction ability of IDV covariance structure was lower than the other residual structures. Overall, model 1 showed better prediction abilities than model 2, mainly due to the strong genomic correlation between the environments. Table 2 summarizes the prediction abilities for the rice dataset using different CV procedures with different models.

**Table 1:** Genomic correlation between the locations on the off-diagonal and SNP-heritability for each location on the diagonal based on models 1 & 3 using the frequentist approach.

	Model 1 (Multivariate)			Model 3 (Univariate)		
Rice	ARK	ABR	FAD	ARK	ABR	FAD
ARK	0.71 (*h*^2^)			0.69 (*h*^2^)		
ABR	0.63 (*r*)	0.47 (*h*^2^)		0.57 (*r*)	0.50 (*h*^2^)	
FAD	0.77 (*r*)	0.65 (*r*)	0.24 (*h*^2^)	0.66 (*r*)	0.45 (*r*)	0.26 (*h*^2^)
Maize	E1	E2	E3	E1	E2	E3
E1	0.58 (*h*^2^)			0.59 (*h*^2^)		
E2	0.54 (*r*)	0.73 (*h*^2^)		0.46 (*r*)	0.73 (*h*^2^)	
E3	0.30 (*r*)	0.11 (*r*)	0.41 (*h*^2^)	0.20 (*r*)	-0.05 (*r*)	0.40 (*h*^2^)

**Table 2:** Prediction abilities (Pearson correlation coefficient between the GEBV and phenotypes) based on five fold cross-validation in the rice dataset.

	*MCMCglmm*			*sommer*		
	ARK	ABR	FAD	ARK	ABR	FAD
**M_CV (Model 1)**						
US	0.68 (0.02)	0.59 (0.02)	0.49 (0.02)	0.68 (0.02)	0.58 (0.02)	0.49 (0.02)
IDH	0.68 (0.01)	0.59 (0.03)	0.51 (0.02)	0.67 (0.01)	0.58 (0.02)	0.50 (0.02)
IDV	0.66 (0.02)	0.56 (0.02)	0.49 (0.01)	–.–	–.–	–.–
ANT1	0.68 (0.02)	0.59 (0.02)	0.49 (0.02)	–.–	–.–	–.–
**S_CV (Model 1)**						
US	0.76 (0.02)	0.69 (0.01)	0.54 (0.01)	0.73 (0.02)	0.67 (0.01)	0.52 (0.01)
IDH	0.77 (0.01)	0.68 (0.01)	0.54 (0.01)	0.75 (0.02)	0.68 (0.01)	0.52 (0.02)
IDV	0.76 (0.01)	0.66 (0.01)	0.53 (0.01)	–.–	–.–	–.–
ANT1	0.75 (0.02)	0.67 (0.02)	0.53 (0.0)	–.–	–.–	–.–
**M_CV (Model 2)**	0.65 (0.03)	0.55 (0.03)	0.43 (0.03)	0.66 (0.03)	0.55 (0.03)	0.44 (0.03)
**S_CV (Model 2)**	0.68 (0.02)	0.61 (0.02)	0.48 (0.02)	0.75 (0.02)	0.60 (0.03)	0.44 (0.03)
**RND (Model 3)**	0.68 (0.01)	0.59 (0.02)	0.48 (0.01)	0.68 (0.01)	0.58 (0.02)	0.46 (0.01)

### Maize data

The maize dataset showed strong G×E interactions (less genomic correlation between the environments (Table 1)) as compared to the rice dataset. Similar to the rice dataset the US, IDH and ANT1 residual covariance structures showed similar prediction abilities. Unlike the rice dataset model 1 & 2 gave the same prediction abilities for both M_CV and S_CV procedures. We believe that this is mainly due to the strong genotype by environment interaction (low genomic correlation between the environments). Table 3 summarizes the results based on different models and CV procedures. The prediction ability based on Bayesian approach was better for the single environment cross validation (S_CV) procedure than the frequentist method. Unlike the rice dataset here we did not find any improvement in prediction ability with S_CV (model 1 & 2) procedure, mainly due to the moderate genomic correlation between the environments.

**Table 3:** Prediction abilities (Pearson correlation coefficient between the GEBV and phenotypes) based on five fold cross-validation in the maize dataset.

	*MCMCglmm*			*sommer*		
	E1	E2	E3	E1	E2	E3
**M_CV (Model 1)**						
US	0.62 (0.02)	0.60 (0.02)	0.47 (0.02)	0.59 (0.02)	0.59 (0.02)	0.47 (0.02)
IDH	0.62 (0.02)	0.60 (0.02)	0.47 (0.02)	0.59 (0.02)	0.59 (0.02)	0.48 (0.02)
IDV	0.62 (0.02)	0.58 (0.01)	0.45 (0.02)	–.–	–.–	–.–
ANT1	0.61 (0.02)	0.60 (0.02)	0.48 (0.02)	–.–	–.–	–.–
**S_CV (Model 1)**						
US	0.65 (0.02)	0.64 (0.02)	0.48 (0.02)	0.61 (0.02)	0.60 (0.02)	0.49 (0.02)
IDH	0.65 (0.03)	0.64 (0.02)	0.48 (0.02)	0.61 (0.01)	0.62 (0.02)	0.49 (0.02)
IDV	0.65 (0.02)	0.61 (0.02)	0.46 (0.02)	–.–	–.–	–.–
ANT1	0.65 (0.02)	0.64 (0.02)	0.48 (0.02)	–.–	–.–	–.–
**M_CV (Model 2)**	0.60 (0.01)	0.59 (0.02)	0.47 (0.01)	0.59 (0.02)	0.59 (0.02)	0.48 (0.01)
**S_CV (Model 2)**	0.64 (0.02)	0.62 (0.02)	0.46 (0.02)	0.60 (0.02)	0.62 (0.02)	0.49 (0.02)
**RND (Model 3)**	0.60 (0.01)	0.59 (0.02)	0.48 (0.01)	0.59 (0.02)	0.52 (0.02)	0.48 (0.02)

## Discussion

In plant breeding, new cultivars are evaluated at several environments and occurrence of genotype by environment interactions (G×E) are common phenomenon in multi-environment trials. One of the main approach to analyze MET data is multivariate mixed model analysis by considering a single trait measured at multiple environments as correlated traits (*e.g*, [12, 13]). In this study, we investigated the impact of four different residual covariance structures on genomic prediction abilities with MET data using multivariate mixed model. Among those four different residual covariance structures, US is the most complex structure with *l*(*l* − 1)/2 parameters (*l* is the number of locations) required to be estimated. But, IDV is the simplest and commonly used covariance structure with a single parameter to be estimated. However, recent studies [18, 30] showed that US covariance structure improve prediction ability compared to the models with IDV or IDH structures. Also our results suggest that the prediction abilities obtained by US, ANT1 or IDH were higher than that of IDV residual covariance structure. Even though, in model 1 the G×E interaction is considered with the genomic covariance matrix (**G**_0_), our results suggest that it is still important to consider the heterogeneous residual covariance structure (US, ANT1, IDH) in genomic prediction of MET data to improve genomic prediction ability.

The univariate model (model 3) performed similarly in most cases to the multivariate model as shown earlier by [8]. However, in presence of a strong genomic correlation between the environments (the rice dataset), the multivariate mixed model (model 1) showed better prediction abilities than the G×E interaction model (model 2). However, when the between environment genomic correlation was low (the maize dataset), both models performed equally well. Thus, our results suggests that multivariate mixed model with heterogeneous residual covariance structure is a preferred choice to account for G×E in genomic prediction of MET data.

The cross-validation scheme of S_CV might correspond to a realistic scenario for breeders where the line is tested in two environments but missing in the third environment. In S_CV procedure the multivariate mixed model (model 1) showed a clear improvement in prediction ability over model 2 for the rice data set. Here the multivariate mixed model, which uses the information from the other tested environments, enhance the prediction ability. Similar findings has been made by [13]. However, with the maize dataset, the S_CV procedure showed relatively low improvement in prediction ability as compared to the rice dataset. This is mainly due to the low genomic correlation between the environments in the maize dataset and the multivariate mixed model cannot borrow information from the other environments in such case.

The Bayesian methods are known to be computationally intensive. However, in this study, our Bayesian approach provided better prediction abilities especially in S_CV procedure, where the observation from a single environment was missing. The slight advantage of Bayesian approach over GBLUP have been already reported by many studies [31, 32]. In GBLUP, the variance components are estimated using REML which is followed by GEBVs. In contrast to that, all parameters are estimated jointly in Bayesian approach and the joint estimation could be one of the factors for the improved prediction ability.

Finally, in this study we only considered the additive genomic relationship matrix (K) for the estimation of the GBLUP values. However, consideration of alternative genomic relationship matrices to better account for different genetic architectures of MET data (*e.g*, [33–36]) in Eq (2) may improve the prediction ability even further. Thus, this is an important topic to be studied in the future.
